# Best practices for acceptability of GM crops field trials conclusions: lessons for Africa

**DOI:** 10.1080/21645698.2024.2376415

**Published:** 2024-07-09

**Authors:** Paul Chege, Julia Njagi, John Komen, Godfrey Ngure, John Muriuki, Margaret Karembu

**Affiliations:** a Program for Biosafety Systems (PBS), International Service for the Acquisition of Agri-biotech Applications (ISAAA AfriCenter), Nairobi, Kenya; b Program for Biosafety Systems (PBS), International Food Policy Institute (IFPRI), Washington, WA, USA; c Inspection, National Biosafety Authority (NBA), Nairobi, Kenya; d Graduate School of Science and Technology, University of Tsukuba, Tsukuba, Japan; e Environmental Science, Kenyatta University, Nairobi, Kenya

**Keywords:** biosafety regulatory, CFT data, decision-making, field trials, GM crops, transportability

## Abstract

The ability to transfer information about the performance, safety, and environmental impacts of a genetically modified (GM) crop from confined field trials (CFTs) conducted in one location to another is increasingly gaining importance in biosafety regulatory assessment and decision-making. The CFT process can be expensive, time-consuming, and logistically challenging. Data transportability can help overcome these challenges by allowing the use of data obtained from CFTs conducted in one country to inform regulatory decision-making in another country. Applicability of transported CFT data would be particularly beneficial to the public sector product developers and small enterprises that develop innovative GM events but cannot afford to replicate redundant CFTs, as well as regulatory authorities seeking to improve the deployment of limited resources. This review investigates case studies where transported CFT data have successfully been applied in biosafety assessment and decision-making, with an outlook of how African countries could benefit from a similar approach.

## Introduction

Most African countries developed their biosafety frameworks under the United Nations Environment Programme’s–Global Environment Facility (UNEP-GEF) project between 2001 and 2004. UNEP-GEF support was initiated to enable countries to establish National Biosafety Frameworks (NBFs) and promote information sharing and collaboration to assist in capacity-building for the domestication of the Cartagena Protocol on Biosafety (CPB). The Cartagena Protocol on Biosafety is a binding international agreement under the Convention on Biological Biodiversity.^1^ The protocol requires countries to establish biosafety procedures for transboundary movement, transit, handling, and use of all living modified organisms that may have adverse effects on the conservation and sustainable use of biological diversity, taking into account risks to human health.^2^ In most jurisdictions, the precedence has been the ratification of CPB, the establishment of national biosafety frameworks, and the enactment of a stand-alone biosafety act with implementing guidelines from which a biosafety regulatory agency is formed and operates.

According to Komen et al.,^3^ concerns in global communities about gene technologies particularly those relating to potential risks to the environment and public health inform strong, sometimes stringent regulatory frameworks that include approval through licensing for biotechnology research, allowing for those risks and concerns to be assessed and managed. The public needs assurance of a low or negligible risk of any adverse reactions to GM food, similar to that of the non-GM comparator. Some communities have voiced concerns regarding the potential occurrence of gene transfer for some traits such as herbicide tolerance from GM crops, thus resulting in herbicide-resistant weeds, and at the same time expressed their fear for “contamination” of traditional crops. Effects of insect-resistant crops on non-target insects or GM crops growing so strong that they become superweeds or pests are some of the other notable concerns constantly voiced by the public. The public is also concerned that some traits such as herbicide tolerance would result in increased use of the chemicals leading to environmental damage.

The regulatory agencies for the technology, anchored in biosafety pieces of legislation, are put in place to ensure the factual distinction between real and perceived risks. As opined by Paoletti et al.,^4^ while the regulatory agencies operate under different legal frameworks, almost all adopt risk assessment strategies that are based on a common set of principles and guidelines. The regulatory agencies are required to conduct environmental risk and food/feed safety assessments before approving the cultivation of GM crop plants, after their development process from experimentation in the laboratory, confined facilities, and field studies.^5,6^

### Environmental Risk Assessments of GM Crops

Data from confined field trials (CFTs) and laboratory testing may be considered during environmental risk assessments of GM crops.^7^ As explained by Glover et al.^6^ and Garcia-Alonso et al.^8^ the CFTs on GM crops must be permitted by the competent regulatory agencies under the country’s biosafety regulatory framework. These trials are conducted under conditions laid out to prevent release of the plant material from the trial site, introgression of the novel trait into populations of sexually compatible species, or the unintended/uncontrolled establishment of the GM plant material into the environment.^8^ The CFTs are conducted in agroecosystems (receiving environments) with a near representation of the ideal environments where the GM crop is cultivated.^8^ In line with the internationally accepted approaches to environmental risk assessment (ERA) of GM plants,^9,10^ a comparative assessment is followed where the GM plant is compared to its conventional counterpart, usually the isogenic or a near-isogenic line, which is included in the CFT as a control. The expected results of such trials depend on the risk hypothesis formulated, but primarily they are supposed to highlight any differences in phenotypic expressions between the GM event and its non-GM comparator as a result of the genetic modification across a range of agro-ecosystems.^9,10^ Control of any elements such as weeds, pests, and diseases that may interfere with the comparison should be considered during the design of the CFTs.^8^

In many jurisdictions, it is a requirement of the national biosafety regulations and guidelines that for any GM crop that is being considered for cultivation approval, in-country field studies to determine any potential environmental risks should be conducted.^11^ Additionally, as pointed out by Slot et al.,^12^ these trials vary per country based on local regulations as there are no international standards for conducting CFTs. It, therefore, follows that irrespective of similarities between growing environments across different countries, CFTs are repeated on a country-by-country basis.^8^ Because of the regulatory conditions in setting up a CFT site, it requires a significant amount of financial, institutional, and human resource investments by both regulatory authorities and product developers, which can present quite a regulatory burden.^8,13^

The repeated CFTs on per country basis regardless of similarities in the growing environments are duplicative and provide no additional, informative data for use in ERA, and this portends a challenge for the public sector and small enterprises with limited resources.^14^ The argument for the acceptability of data – data transportability – from CFTs conducted in one country, a so-called remote country, to be relevant and sufficient in a local country for ERA regulatory purposes can therefore be considered reasonable.^15^ One-way data transportability can be plausible if the agro-climatic conditions of the location where the CFT is conducted are demonstrably representative of the agro-climatic conditions of the area in which the GM crop is being considered for cultivation.^8^ In some jurisdictions, for data to be transportable, the novel traits need to exhibit “familiar traits”- the mode of action (MOA) needs to be thoroughly understood as evidenced by a peer-reviewed publication or a national investigative commission and the efficacy of the trait being assessed needs to be comparable to that of the other traits which have already been approved.^11^ It has also been posited that regulatory data requirements should be risk hypothesis-driven and conducted within the ERA framework that takes into account adequate problem formulation.^7,16^ The regulators often require that the GM crop plant does not exhibit different growth under different environmental conditions such as soil type and weather conditions. Additionally, various jurisdictions require a clear understanding of the GM crops’ conventional counterparts’ agro-morphological performance within the country, the general weediness potential of a crop, and the presence of sexually compatible wild relatives of the crop in the country.

### Food Safety Assessment of GM Crops

Presently, most of the principles of food safety assessment are drawn from the Principles for the Risk Analysis of Foods Derived from Modern Biotechnology (Principles Document), Guideline for Safety Assessment of Foods Derived from Recombinant-DNA Plants (Plant Guideline), and Guideline for Safety Assessment of Foods Derived from Recombinant-DNA Microbes.^17^

As explained by Paoletti et al.,^4^ the Principles Document discusses risk assessment, risk management, and risk communication and describes the safety assessment as a component of the risk assessment. The essence of the safety approach is that the new food (or component thereof) should be compared with an appropriate conventional counterpart, that is, with food already accepted as safe based on its history of safe use as food. The assessment should follow a structured and integrated approach. It should evaluate both intended and unintended effects, that is, intended and unintended differences from the conventional counterpart; it should identify new or altered hazards; and it should identify any changes in key nutrients that are relevant to human health. The guidance provides that such a safety assessment of food derived from a recombinant-DNA plant follows a stepwise process of addressing relevant factors that include i. description of the recombinant-DNA plant, ii. description of the host plant and its use as food, iii. description of the donor organism(s), iv. description of the genetic modification(s), v. characterization of the genetic modification(s), vi. safety assessment, and vii. other considerations. On safety assessment, details on the expressed non-nucleic acid substances, compositional analysis of the key components, evaluation of metabolites, food processing, and any nutritional modifications are considered. The incorporation of food safety assessment data strengthens the scientific basis for CFT data transportability and builds confidence in acceptance of the data by the regulatory authorities of the receiving countries about the safe use of the GM crop in question.

## Key Considerations in the Transportability of Data

As already pointed out and as Garcia-Alonso et al.^8^ describe, field trials’ measurable endpoints vary, depending on the risk hypotheses being tested, but most of these studies are designed to identify differences between the transgenic crop and its non-transgenic counterpart, resulting from intended or unintended consequences of the genetic modification, across a range of agroecosystems. The measurable endpoints in the CFTs that inform the study are crop-specific and generally encompass those characteristics relevant to plant emergence, vegetative growth, and those related to the reproductive biology of the plant.^14^

In the CFTs, biological differences emanating from the novel gene insertion are assessed under highly controlled conditions within crop production zones in different agro-climatic and agro-ecological conditions.^14^ These differences as Vesprini et al.^14^ explain are deduced from comparative assessments between transgenic and non-transgenic crops, grown side by side in the same environment and subject to the same agronomic management practices. Best practices in the assessment of these differences are that CFTs should be conducted in different environments, as long as they cover a range of environmental conditions within the country/region. Therefore, for a given crop, the CFT data obtained are universal and may be accepted in remote countries unless in a unique situation where the risk hypothesis for a particular receiving environment cannot be addressed by the available data, necessitating a local CFT to generate the new required information.

According to Vesprini et al.,^14^ on analysis of the various examples of CFT data that have been accepted in remote countries, even when climate and production practices vary, the environmental safety conclusions from the comparative assessments are consistent across geographies provided that studies are run across a broad range of conditions. Various studies including^11,15,18–22^ among others have reported transportability of data generated in different geographies for the ERA of GM soybean and maize. Risk assessment of GM soybean MON 89788 vis-à-vis its conventional counterpart A3244 across the United States and Argentina showed no effects on weed characteristics and ecological impact attributable to the GM trait.^18^ Similarly, using 14 sites across the United States, Argentina, and Brazil, Ahmad et al.^15^ reported no adverse impact of GM maize MON 87411 on non-target arthropods compared to its conventional counterpart. By comparing agronomic CFT data from 11 GM soybean events in Japan, and comparative data generated in the United States, Matsushita et al.^22^ demonstrated the utility of transported CFT data to inform ERA of GM crops in Japan. This overwhelming experiential evidence negates the need for replicating CFTs in every country or region intending to release a transgenic, as doing so would not lead to new/different conclusions from those obtained from remote CFTs, even though new data may be added to the already existing data.

Vesprini et al.^14^ advise that for the assessment of conclusions from CFT studies, appropriate experimental design and methodologies need to be put into consideration besides the relevance and consistency of the measured endpoints across the studies being upheld. Also important in the assessment of such conclusions for acceptability in remote countries is the diversity of the agroecological zones of the crop-growing zones in which the CFTs were carried out.

According to Vesprini et al.,^14^ the concept of substantial equivalence provides a basis to determine if the foods/feeds derived from a transgenic plant are as safe as their conventional counterparts^17,23,24^ from the food and feed safety assessment perspective. Key nutrients, anti-nutrients, secondary metabolites, and toxins for both the genetically modified crop and its conventional counterpart comparator are the typical endpoints in the compositional studies.^24–27^ The edible plant parts are harvested from the CFTs to provide the samples for the compositional analyses.

For the acceptability of remote data on compositional assessment, Codex guidelines^17^ among other documents provide a reference framework. In addition, for the identification of the relevant components for a specific crop in a comparative analysis, OECD consensus documents on the composition of crops, containing key nutrients, anti-nutrients, and toxicants^28^ have been widely utilized.^14^ Other databases on crop composition which display ranges of natural variability that have been established from diverse global sources and seasons for conventionally bred commercial cultivars such as the Agriculture & Food Systems Institute^29^ are also widely consulted.

## Case Study of Data for Virus-Resistant Transgenic Bean Developed in Brazil Accepted in Argentina

This study seeks to borrow insights from the case study of the data for virus-resistant transgenic bean developed in Brazil that was accepted in Argentina and therefore explore whether the criteria that informed the acceptability of the data could be applicable to other crops’ data and countries. The criteria as explained by Vesprini et al.^14^ comprised three conditions, namely, the application of appropriate experimental design and methodologies; relevance and consistency of the measured endpoints; and diversity of the agroecological locations where the trials were conducted.

Vesprini et al.^14^ explains that the transgenic bean line named “Embrapa 5.1” was compared with the conventional parent line named “Olathe” in a randomized complete block design trial with eight replications in different locations over 2 years. The trials were treated to the general agronomic management typical for a bean crop production system that included fertilizer applications following soil tests in a particular location, irrigation, herbicide, and insecticide applications. Any non-trait-related differences in pest pressure as well as in the crop’s agronomic performance among the trial plots in a given site were minimized by applying the same management uniformly across all plots at each site. The effects of treatments on each site were determined by Analysis of Variance (ANOVA) using Statistical Analysis System (SAS) software with the analyses being carried out over random factors location/year for each location with *p* < .05 as the level of significant differences.

The compositional studies of the transgenic bean benefited from a *de novo* common bean composition database developed by the Brazilian Agricultural Research Corporation (EMBRAPA) that was developed after growing common bean varieties over 5 years in multiple locations, ensuring a range of natural variations for each analyte. The compositional database is now part of the OECD consensus document on compositional considerations for common bean.^28^

Regarding the relevance and consistency of the measured endpoints, the parameters selected for the study were considered for appropriateness and sufficiency for risk characterization: they adequately reflected the common bean agro-morphological characteristics that are critical for common bean productivity. These parameters according to Vesprini et al.^14^ comprised of yield, seedling emergence, seedling height, the maximum width of the primary leaves, maximum length of the primary leaves, number of seeds per pod, the weight of 100 seeds, pod length, pod width, seed length, seed width, the thickness of seeds and flowering time.

As for the compositional analysis, the ERA study for the Embrapa 5.1 transgenic bean and its comparator comprised endpoints considered for the analysis in raw and processed (cooked) beans including carbohydrates, vitamins B1 and B2, minerals, amino acids, and proximates, as well as anti-nutrients phytic acid and trypsin inhibitors. The analytes are included in the recommendations of the OECD Consensus Document for common beans.^14^

Lastly, and as parts of the three sets of criteria for which conclusions for the transgenic bean from Brazil were considered transportable for approval in Argentina, the diversity of the crop production areas where the CFTs were conducted was considered. The diversity of the environmental conditions was assessed by considering the geographical locations of the sites (latitude/longitude), historical water balance, and other environmental factors such as temperature, humidity, and precipitation.^14^ Although Garcia-Alonso et al.^8^ opines that soil type is not a key parameter for data transportability, it was taken into consideration as a secondary element to distinguish environments. Vesprini et al.^14^ report that the CFTs’ locations covered different production zones and that when wholesomely observed, the characteristics evaluated showed agronomically relevant differences between locations.

In the end, the CFTs for the transgenic bean and the conventionally bred counterpart showed no biologically relevant differences. Vesprini et al.^14^ report that the few statistically significant differences found for the measured endpoints were not consistent across locations or years in a particular location. The difference could be considered random and not occasioned by either a specific location or the gene insertion: the transgenic bean and its conventional comparator could be considered in terms of composition, nutritionally, and agro-phenotypically equivalent.

These criteria for evaluation of a given crop’s CFT conclusions acceptability in remote countries should be adopted and widely practiced in Africa. This would encourage expedited adoption of highly required improved crop plants to be adopted in African countries where crops resistant to endemic and invasive pests and diseases and those that are resistant to climate change-related challenges such as drought or flooding, and unpredictable weather changes are urgently needed. Data transportability would enhance investment by low-budget projects and companies, as it would eliminate the need for the expensive and unnecessary repeat of risk evaluations in confined trials.

## Assessment of the Suitability of African GM Approvals for Data Transportability

There exists a considerable wealth of knowledge from close to three decades of creating, selecting, and breeding GM crops, which indicates that these crops are no more likely to have harmful unintended effects as compared to other methods such as mutagenesis, hybridization, working at introducing genetic variation into crops.^30,31^ Particularly, Africa rounded the 25th year of commercial cultivation of biotech crops in 2022, with four crops, maize, cotton, soybean, and cowpea approved in seven countries. The insect-protected maize, virus-resistant cassava in Kenya, and insect-protected cowpea in Ghana have received approval for environmental release. A total of 23 countries have enacted biosafety laws with 14 of those actively conducting confined field trials (CFTs). Uniquely, Uganda has had the largest number of CFTs even though it is yet to enact a stand-alone biosafety law. As demonstrated by the accelerated pace in the number of commercial approvals in Nigeria after the enactment of the National Biosafety Management Agency Act, 2015 and considering that Uganda has not had any commercial approvals yet, there is a positive correlation between the adoption of biosafety laws and the pace in placement on the market of biotech crops. Biosafety regulatory data transportability has not been widely embraced in Africa with the countries that have adopted GMOs requiring CFTs as part of the ERA process. In Kenya for instance, it is a requirement that three CFTs are carried out for a year or one CFT for 3 years.^32^ However, the Kenyan biosafety law has a provision for data transportability (Section 28) in instances where there is sufficient information to conclude that GMOs under review for import, research, or environmental release do not pose a significant risk.^33^ Kenya’s biosafety authority has in the past considered data generated elsewhere as part of risk assessment for import and transit of GMOs for food, feed, and processing (http://ke.biosafetyclearinghouse.net/importandtransit.shtml). Other African countries utilizing data transportability in their decision-making process for imports of GMOs for food, feed, and processing include Ghana and Nigeria.

This segment seeks to analyze the approved crops’ data acceptability in remote countries, based on the above-said criteria.

### Bt cotton

According to Endale et al.,^34^ Bt cotton had been approved for commercial cultivation in South Africa, Nigeria, Sudan, Ethiopia, Kenya, Malawi, and Eswatini by 2022. In these seven countries, Bt cotton has been adopted because it offers an effective and inexpensive way to control damage from African bollworm (*Helicoverpa armigera*) and other insects that frequently damage cotton in Africa.^35^ The Bt cotton varieties in all these countries express the Bt protein produced by a ubiquitous soil bacterium (*Bacillus thuringiensis*) which when ingested by an insect, the digestive system activates a toxic form of the Bt protein and kills the target insect within a few days.^36^ The seven countries have had a long tradition of cotton growing, predominantly by smallholder farmers who before the commercialization of Bt cotton had continued to grapple with several challenges including the high cost of labor; minimal use of necessary inputs for intensification (e.g., fertilizer, herbicides, etc.); inadequate availability of quality seed; unstable and low seed cotton prices paid to farmers; and most importantly, pest damage, particularly by the African bollworm. In the African countries that would explore data transportability on Bt cotton, there is a clear understanding of Bt cotton’s conventional counterpart’s agronomic performance, therefore meeting one of the conditions put across by Nakai et al.^11^ as earlier explained. Cry1Ab expressed by Bt cotton and similar proteins have been used extensively in GM crops worldwide, providing benefits to farmers, consumers, and the environment in both developed and developing countries.^37–39^ The environmental and health risks attributed to crops producing these proteins are well characterized and negligible.^40,41^ This paper opines that if the agro-climatic condition of the source country is representative of that of the remote country, data from Bt cotton CFTs can be reliably exchanged as the establishment of a new CFT does not lead to any new/unique data.

### Bt maize

According to Van den Berg et al.,^42^ maize is a major food security crop for populations in sub-Saharan Africa (SSA), comprising 46 countries. As such, the crop has been grown in this region for a long time, conferring a long tradition of cultivating the crop. This has, however, not been without accompanying pest and disease challenges, more recently, the invasion of the fall armyworm, *Spodoptera frugiperda* (J. E. Smith) (Lepidoptera: Noctuidae),^42^ among other pests including maize stalk borer (*Busseola fusca*).^43^ Several authors have advanced solutions, including the use of Bt maize, genetically engineered to produce insecticidal proteins to limit yield losses.^44,45^ Bt maize has been cultivated in South Africa since 1998,^46^ and globally by more than 14 countries since 1996, representing 29% of all maize grown.^42,47^ In several other African countries, including Ethiopia, Kenya, Mozambique, Nigeria, and Uganda, at various stages in the crop’s approval for open field cultivation, the Bt maize varieties containing mainly the MON 810 event are sufficiently efficient against the lepidopteran pests mentioned.^48,49^ In addition, various studies have found the Bt maize to be safe for human consumption and the most beneficial to arthropods, comprising pollinators and natural enemies of pests.^42,45^ Based on the regional and global experience in research with Bt maize, this paper avers that data could be transferrable in the region if the experiments in the source country have applied the appropriate experimental design and methodologies, the measured endpoints are relevant and consistent, and they have been conducted in diverse agro-ecological locations. Additionally, if the countries have demonstrably similar agro-climatic conditions for maize cultivation, a new CFT would not yield any different data, negating the need for investment.

### Virus resistant cassava

In 2021, the National Biosafety Authority (NBA) in Kenya granted limited general-release approval of GM cassava event 4046 developed under the Virus Resistant Cassava for Africa (VIRCA) project for the purposes of conducting National Performance Trials (NPTs). This was based on data generated both under greenhouse and confined field trials at locations known to have very high Cassava Brown Streak Disease (CBSD) pressure to evaluate the level of resistance of event 4046.^50^ The data, which was generated from field trials established at Namulonge, Uganda, and Mtwapa, Kenya, contributed to the approval decision in Kenya, exemplifying the possibility of data transportability. Essentially, the approval decision in Kenya was the first on a cassava crop globally. Through a collaborative program between the Donald Danforth Plant Science Center, USA, the National Crops Resources Research Institute, Uganda, and the Kenya Agriculture and Livestock Research Organization, Kenya, the cassava plants were improved through RNAi silencing to confer resistance to CBSD.^51^

Evaluations were carried out over two cropping seasons at Namulonge, Uganda, and a single season at Mtwapa, Kenya. Event 4046 consistently demonstrated a high level of resistance to CBSD at both locations, consistent with its predicted performance based on high levels of siRNA expression.^52^ Trials in both Kenya and Uganda were established in a Randomized Complete Block Design, each evaluating the transgenic cassava together with its conventional counterpart. In both CFTs based in Namulonge and Mtwapa, plants were visually assessed for CBSD symptoms beginning 1 month after planting and every month thereafter. The severity of CBSD symptoms on shoots and stems of plants within each plot was assessed and scored as described by Ogwok et al.^53^ Incidence was calculated as the percentage of plants showing cassava brown streak disease symptoms on leaves and stems per event.

In the multi-year analysis, there were no statistically significant differences between event 4046 and control TME 204 cassava in any of the agronomic parameters measured at harvest, including plant height, plant branch height, above-ground biomass, stems per plant, marketable roots per plant, root weight per plant, marketable root weight per plant, and harvest index. The growing season (year) was a significant factor for some phenotypic parameters, such as plant height, above-ground biomass, marketable roots per plant, root weight per plant, and harvest index. In these cases, where there was a significant year-to-year variation, there were no significant genotype-by-year interactions, indicating that relatively, the two genotypes were consistent for the traits across years.

The comparative agronomic and phenotypic data support the conclusion that the genetic modification resulting in event 4046 did not have an unintended, unexpected effect on plant growth habit and general morphology, vegetative vigor, or root yield that would indicate any risk to the environment. From the data and observations, there were no indications that 4046 cassava would be more invasive or persistent in the environment or have altered susceptibility to insect pests, and bacterial and fungal diseases, compared to conventional cassava. On the contrary, due to genetic modification, transgenic cassava does not require spraying against pests that spread the CBSD virus and this is beneficial to non-target faunal species including general environment health, including and that of farm workers. Cassava plants are almost always propagated by vegetative means in farm fields. In warmer tropical areas, the crop tends to have weak flowering and seed formation systems, very sensitive to environmental factors, thus making the chances of hybridization or outcrossing with wild varieties minimal. With respect to plant growth and development, morphology, and yield, event 4046 cassava was found to be equivalent to conventional cassava.

This paper puts forward that the trial data on the CBSD-resistant cassava with event 4046 was conducted in a manner that allows tracking of all the aspects outlined by Nakai et al.^11^ and Vesprini et al.,^14^ and therefore it is amenable for data transportability to any receiving country in the region. Additional data obtained through local trialing should only be warranted in instances where there is a plausible pathway to harm on a valued entity identifiable through problem formulation.^7^

## Policy recommendations and conclusion

Africa, especially sub-Saharan Africa (SSA), continues to witness the highest number of food insecure people globally. Many studies have projected a worsening situation, judging by productivity trends for cereals and roots and tuber crops’ performance in the last 50 years. This is despite the continent adopting blueprints such as the United Nations’ Sustainable Development Goals (SDGs), which aspire to end hunger, achieve food security and improved nutrition, and promote sustainable agriculture by 2030 (SDG2), and the Malabo declaration that promised to end hunger and halve post-harvest losses in Africa by 2025. These challenges call for newer technologies that promise substantive intensification (increased production on the same or fewer resources), to ease the pressure on land, and assure improved farmer well-being and incomes, thus attracting youths to crop production.

The adoption of agricultural biotechnology in many parts of the world over the last three decades has demonstrated the technology’s contribution toward increased productivity, self-sufficiency on a nation’s arable land, biodiversity conservation, climate change challenges mitigation, improved health, social, and economic benefits. While many African countries outside of South Africa have missed out on these benefits, the domestication of the Cartagena Protocol on Biosafety (CPB), adoption of biosafety and biotechnology policies and legislation in many of these countries is testament to the desire to safely apply agricultural biotechnology.

Risk assessment studies come with opportunity costs and this may be a deterrent to the adoption of beneficial products. Therefore, requests for additional regulatory data are not always justified. A critical consideration must be made where there is demand for additional testing of GM activities that pose low risk based on a proven long history of safe use and where additional testing has often failed to contribute significantly to the assessment process. Hence, calls for additional testing of GM crops with a solid safety track record should be examined critically to determine whether the value of additional testing outweighs the costs. The need for biosafety studies cannot be determined solely by scientific analysis, but rather a judgment by local regulators based on their priorities, which may differ among countries. Ultimately, decisions to require further studies may be made for reasons other than the scientific risk assessment that will have been done in the source countries.

This paper sought to explore one of the ways through which the adoption of agricultural biotechnology can be realized without undue lengthy periods in the attainment of requisite biosafety compliance if data on a biotechnology product exist and could be transferrable across borders. Several scholars have come up with a consensus on the conditions necessary for data transportability. They argue that for data to be transportable, the agro-climatic conditions of the location where the CFT is conducted should be demonstrably representative of the area to which the crop is being considered for cultivation. The scholars point out that there needs to be a clear understanding of the biotech crop’s conventional counterpart’s agro-morphological performance within the country to which the data is being transported attained through a long tradition of growing the conventional varieties. They also recommend that for data to be transportable, the novel traits in the biotech crop need to exhibit familiar traits, i.e., the mode of action needs to be thoroughly understood as evidenced by peer-reviewed publications. The efficacy of the new trait needs to be comparable to that of similar traits that may have already been approved elsewhere. Additionally, the CFTs conducted in the source country should have been conducted in diverse agroecological localities, employing scientifically sound, replicable methods, and using relevant and consistent measure endpoints. The study analyzed some of the biotech crops approved for general environmental release in Africa, namely Bt cotton, Bt maize, and CBSD-resistant cassava. It found that the conduct of the respective CFTs in various countries was in a manner allowing CFT data to be transportable, based on the demonstrated safety of the GM crops, and the receiving country or countries meeting the condition of having a demonstrable representative agro-ecological zone compared to the source country. Data transportability should be applicable to reduce biosafety regulatory burden in terms of costs and time, where additional CFTs may not generate new data to inform decision-making.
